# In Vitro Models for Improved Therapeutic Interventions in Atrial Fibrillation

**DOI:** 10.3390/jpm13081237

**Published:** 2023-08-08

**Authors:** Jara M. Baena-Montes, Marcin J. Kraśny, Martin O’Halloran, Eoghan Dunne, Leo R. Quinlan

**Affiliations:** 1 Physiology and Cellular Physiology Research Laboratory, School of Medicine, Human Biology Building, University of Galway, H91 TK33 Galway, Ireland; 2 Smart Sensors Lab, Lambe Institute for Translational Research, School of Medicine, University of Galway, H91 TK33 Galway, Ireland; 3 Translational Medical Device Lab (TMDLab), Lambe Institute for Translational Research, School of Medicine, University of Galway, H91 TK33 Galway, Ireland; 4 Electrical & Electronic Engineering, School of Engineering, University of Galway, H91 TK33 Galway, Ireland; 5 CÚRAM SFI Centre for Research in Medical Devices, University of Galway, H91 TK33 Galway, Ireland

**Keywords:** atrial fibrillation, cardiac models, screening platform, in vitro

## Abstract

Atrial fibrillation is the most common type of cardiac arrhythmias in humans, mostly caused by hyper excitation of specific areas in the atrium resulting in dyssynchronous atrial contractions, leading to severe consequences such as heart failure and stroke. Current therapeutics aim to target this condition through both pharmacological and non-pharmacological approaches. To test and validate any of these treatments, an appropriate preclinical model must be carefully chosen to refine and optimise the therapy features to correctly reverse this condition. A broad range of preclinical models have been developed over the years, with specific features and advantages to closely mimic the pathophysiology of atrial fibrillation. In this review, currently available models are described, from traditional animal models and in vitro cell cultures to state-of-the-art organoids and organs-on-a-chip. The advantages, applications and limitations of each model are discussed, providing the information to select the appropriate model for each research application.

## 1. Introduction

Atrial fibrillation (Afib) is the most common type of cardiac arrhythmia in humans, characterised by high-frequency excitation of the atrium, which results in dyssynchronous atrial contraction and irregularities in ventricular excitation [1]. Afib is increasingly a widespread health problem as its prevalence is on the rise, with an estimated 33 million people worldwide diagnosed with Afib in 2020; this figure is expected to double by 2050 [2]. Afib patients have increased morbidity and mortality rates due to the severe consequences associated with thromboembolism and stroke [3]. Ischemic stroke and Afib commonly exhibit overlapping risk factors, including dyslipidemia, diabetes mellitus, and hypertension. Oral anticoagulants are widely prescribed for stroke prevention in patients with Afib. Nevertheless, it is noteworthy that these anticoagulants solely address the thromboembolic aspect and do not comprehensively target the entirety of factors associated with Afib pathology [4]. Moreover, COVID-19 infection is reported to be associated with more frequent occurrences of arrhythmias, predominantly Afib cases [5], making Afib a global concern that needs more efficient and effective treatment options.

**Figure 1:**
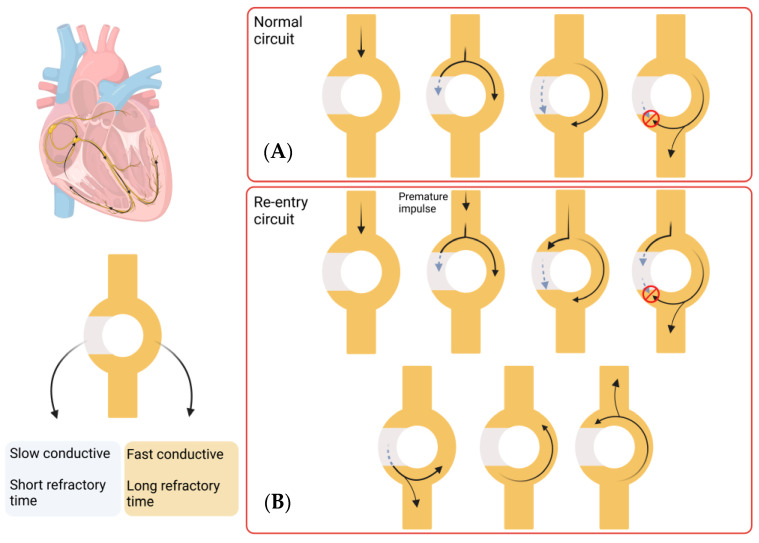
Re-entry process in Afib. Most of the re-entry circuits happen above the level of the ventricles, but this process can take place in any area of the heart. This circuit consists of a single pathway that divides in two, one which shows slow conductivity and short refractory time and the other which shows fast conductivity and longer refractory time. (**A**) In a normal circuit, an impulse travels through the circuit and splits into both pathways. The fast conductive pathway propagates the impulse quicker, but the long refractory period makes it inaccessible for a long time (red circle indicates the pulse cannot propagate). The slow conductive pathway propagates the impulse slower, with a quicker recovery. When the impulse in the fast conductive pathway reaches the next pathway, it splits again and propagates the impulse, while the other component interacts with the slow conductive pathway impulse and annulates each other. In this way, the impulse is transmitted forward. (**B**) A re-entry circuit is created by a premature impulse, which would take the slow conductive pathway due to the short refractory time. This leads to an impulse that does not fade and persists in propagating impulses in both directions, leading to a continuous re-excitation of the heart. (Images created with BioRender.com (accessed on 21 January 2023)).

The pathophysiology underlying Afib involves a complex process that is not yet fully understood. Clinically, Afib is divided into paroxysmal, persistent, long-standing persistent, or permanent, depending on the duration of the symptoms. Paroxysmal Afib typically resolves within 7 days of onset, while persistent Afib is sustained for longer than 7 days. Long-standing persistent Afib lasts more than 12 months, and Afib is considered permanent when there has been a joint decision by the patient and clinician to cease further attempts to restore or maintain sinus rhythm [6]. A key component in the maintenance of the Afib arrhythmia state is the process of re-entry, which occurs when an impulse travels abnormally around a cardiac circuit repetitively. This is a triggered process that initiates an arrhythmia, which is commonly induced by an ectopic firing focus. The first reported focal ectopic firing loci were found in the pulmonary veins (PVs) in patients with paroxysmal Afib; ablation of these ectopic foci was shown to reduce Afib burden, demonstrating a role for PVs in Afib genesis [7]. Several subsequent studies have provided evidence for the role of PVs in Afib initiation [8,9,10]. These processes are defined as the group of molecular, cellular and interstitial changes that can manifest as changes in size, mass, geometry and function of the heart after injury [11]. Some of these changes include progressive atrial dilatation [12] or changes in ion channel activity, (particularly Na+ and K+ currents) [13]. Overall, this results in a vulnerable tissue substrate that facilitates the process of re-entry that maintains Afib. Re-entry itself is a process that occurs when a propagating impulse fails to fade after normal activation of the heart and persists, resulting in continuous re-excitation of the heart even after the refractory period has ended. Re-entry requires some type of stimulus that triggers a vulnerable circuit (tissue substrate) where the depolarising signal never encounters refractory tissue which results in the typical increase in heart rate of arrhythmic patients [14]. A schematic representation of this process is shown in Figure 1.

### 1.1. Current Therapeutic Options for Atrial Fibrillation

The main therapeutic goal in the management of Afib is the restoration and maintenance of a sinus rhythm. Anti-arrhythmic drugs (AADs) remain the most widely prescribed treatment for Afib patients; however, they are not always effective and show significant adverse effects, including some that for certain patients are pro-arrhythmic [15]. Anti-arrhythmic drugs typically act on ion channels, with the main goal of reducing the frequency and duration of episodes of arrhythmias. AADs are classified depending on the mechanism of action and specific effects on the heart. Class I AADs are sodium channel blockers, and they reduce the conduction velocity and are commonly used to treat atrial and ventricular arrhythmias. They are further subdivided into IA (such as quinidine or procainamide), IB (such as lidocaine or mexiletine) and IC (such as flecainide and propafenone). Class II AADs are beta-blockers, and they stop epinephrine and norepinephrine from binding to the beta-adrenergic receptors, decreasing conduction through the AV node. Some examples are propranolol and metoprolol. Class III AADs block potassium channels, repolarising the cells and prolonging the action potential duration. The most used are amiodarone, sotalol and dronedarone [16,17]. However, the positive effects of these drugs are accompanied by increases in mortality risk in long-term treatments and, on some occasions, exacerbation of the arrhythmia that they are meant to treat [15,18]. In this review, we will focus on non-pharmacological treatment approaches with a specific focus on the utility of in vitro models in supporting the development and enhancement of catheter-based ablation techniques.

### 1.2. Catheter Ablation

Catheter ablation aims to isolate, disrupt or possibly destroy abnormal foci responsible for Afib. The four myocardial sheaths of the left atrium arising from the pulmonary veins (PV) are identified as the primary trigger source in 82% to 90% of patients with paroxysmal atrial fibrillation. Therefore, PVs are the most common anatomical structures targeted by catheter ablation in the treatment of atrial fibrillation [3]. Some evidence also supports the idea of ablation targeting the ostium of the left atrial appendage (LAA) due to the prevalence of abnormal firing in patients with recurrent Afib [19]. Currently, for Afib, the treatment falls under a thermal or non-thermal ablation. However, recently, the non-thermal approach of pulsed-field ablation (PFA) has been gaining prominence [20]. The most common thermal method is in the form of radiofrequency ablation (RFA), followed by high-intensity focused ultrasound (HIFU) or cryoablation. Currently, catheter ablation is now considered the first-line treatment strategy for symptomatic paroxysmal atrial fibrillation [21]. 

Radiofrequency ablation uses heat to destroy the area of tissue triggering the Afib. Reported Afib treatment using RFA shows higher efficacy than AAD therapy, with reduced complications [22]. Despite increasing adoption in the clinic, the success rate with RFA varies depending on the patient and the type of Afib present. For the ideal candidate receiving ablation therapy, presenting for paroxysmal Afib, the effectiveness is between 60 and 80% [23]. However, for less-than-optimal patients, such as a patient with persistent Afib, the success rate lies between 50 and 70% [23].

High-intensity focused ultrasound is based on the mechanism of vibration that produces mechanical movement of particles within a medium, which is then converted to heat causing thermal tissue injury. HIFU creates wide and deep lesions (up to 11 mm in depth) and it is energy dose-dependent [24]. HIFU has been shown to have the ability to precisely target a defined location, for example, creating lesions in the cardiac tissue while ensuring that the epicardium and endocardium remain unaffected [25]. In comparison to cryotherapy or RFA, HIFU can easily penetrate soft tissues and can produce an ablation region without the need for direct physical contact. This feature makes HIFU advantageous in situations where a challenging location of the tissue impacts the stability and consistency required for tissue contact. Limitations to HIFU include reported permanent injuries to extracardiac tissue such as the wall of the oesophagus and the phrenic nerve leading to nerve palsy and dyspnoea [26].

Cold ablation or cryotherapy is characterised by the formation of intracellular and extracellular ice crystals, which results in apoptosis of the surrounding tissue. These crystals are produced by cryothermal energy that is produced by the injection of refrigerant through a fine tube catheter, freezing the tissue. The refrigerant vaporises at the tip of a cryoablation catheter and freezes the tissue [27]. While freezing, the catheter tip adheres to the affected tissue, which enables the application of stable energy delivery. In contrast to RFA, tissue lesions induced by cryoablation maintain the tissue structure, including fibrocytes and collagen [28] and present a lower risk of cardiac perforation or thrombogenicity compared to RFA [29]. However similarly to RF, cryo-based ablations can result in damages to the oesophagus [30].

The latest emerging therapy for Afib is pulsed-field ablation. The PFA modality is a non-thermal catheter-based ablation technology that uses high-voltage pulsed electrical fields to ablate tissues through a mechanism known as irreversible electroporation (IRE). This process is based on the application of high electric fields to a cell, leading to the formation of large pores in the cell membrane, ultimately resulting in cell death. IRE has been used previously in other medical areas such as oncology, and due to the reported increased tissue specificity compared to other approaches, PFA may present a safer catheter-ablation option compared to RFA [31] or cryoablation [32]. 

### 1.3. Limitations of Current Treatment Approaches

Current therapeutic options are expanding and improving to maximise efficiency and reduce adverse effects on the patient. However, the extensive number of potential targets, variables and parameters associated with the ideal catheter-based intervention creates a challenge in achieving maximally successful control of Afib. The current range of applied parameters may contribute to the variable successes reported in the clinic. To facilitate the development of an experimental-based set of parameters and to achieve successful treatment at minimal cost, it is essential to design an appropriate testing platform that allows us to refine parameters as well as predict and mitigate potential negative side effects. There is a need to strike a balance between the testing and optimisation of the treatments while avoiding the risk to the life and well-being of the patients. The development of appropriate models of Afib is essential not only as a disease model to better understand the mechanisms underlying Afib but also as a therapeutic screening platform to facilitate the testing and development of different treatment options. The absence of appropriate and well-defined models for Afib for in vitro and preclinical testing can be an obstacle to developing improved therapeutics. A good model should allow the testing of a broad spectrum of treatments, which would result in the selection of the best option to be included in future clinical trials. Without these models, only limited information can be obtained, and important side effects can be missed [33,34]. In recent years, several research models have been developed to mimic human cardiac atrial fibrillation, each of which can provide different information depending on their advantages and limitations. In this review, we report on the different models available for the study of Afib, highlighting their advantages and disadvantages, relevant publications/outcomes and most common uses. 

## 2. Current Experimental Models of Atrial Fibrillation

When choosing an experimental model of Afib in the context of device ablations, it is important to consider the level of complexity that is required to answer the experimental question being asked. Animal models provide the highest level of complexity (most commonly dogs, pigs and horses), which provide insight into heart function, effects on surrounding tissues, other organ systems and behavioural responses to interventions. Ex vivo isolated heart studies offer the opportunity to analyse tissue mechanisms under controlled conditions which are very relevant for local short-term acute effects. Finally, the use of in vitro cellular models, both in single-cell monocultures of cardiomyocytes or in the creation of multi-layered and multi-cellular cardiac tissues provides insight into the cellular and molecular mechanisms involved in Afib processes and the effect of the potential treatments, allowing the specific study of changes in cell death and beating patterns. As the complexity of the model increases, typically, the opportunity for higher throughput testing decreases. Thus, the development and optimisation of treatments require a balanced approach and planning a pipeline of tests across multiple levels. Different in vivo animal models are currently available and show many similarities to human Afib studies in terms of benefits but also limitations in terms of costs and ethical considerations. While the data from animal studies have provided valuable information on Afib and its pathophysiology and recent research has successfully induced Afib in animal models [35,36], our understanding and the development of treatment options and clinical targets were revealed through several clinical studies in humans. For instance, Haïssaguerre et al. reported that Afib is initiated by focal areas near the pulmonary veins (PVs) [7]. Furthermore, they showed that RFA of the area surrounding the PVs, so-called pulmonary vein isolation (PVI), was sufficient to terminate Afib [37]. The focus of this review is ex vivo and in vitro models of Afib, as animal models have been extensively reviewed elsewhere [38].

### 2.1. Ex Vivo Models

Cardiac tissue isolated from animal hearts has been widely used for cardiac studies using a range of biochemical, electrophysiological, pharmacological and morphological approaches, particularly related to antiarrhythmic drug safety [39,40]. Availability of human cardiac tissue is, in general, limited to very small amounts of fresh and viable sections which are considered surgical waste [41,42]. However, these small samples do contribute to the study of the mechanisms and effects of Afib therapy [43,44,45]. Human-explanted tissues help to overcome some of the physiological differences seen in animal models. Nonetheless, the amount and complexity of the available tissue are usually insufficient, often lacking the multi-layered structures which make them closer to conventional in vitro models. These models have the great advantage that they avoid any interference from the autonomic nervous system and internal organ communications, which allows the examination of pure cardiac responses against different interventions. The use of ex vivo cardiac models to determine the ablated area produced by a certain ablation modality has the potential to allow for the testing of a wide array of experimental parameters [46,47,48]. Relevant mechanical and physical aspects of catheter ablation, such as contact angle or contact force, can be optimised for an optimal ablation outcome by ex vivo models as previously reported [49]. Similarly, the effect of temperature or the irrigation solution used in the RF ablation process can be easily tested and measured in excised porcine ventricles [50].

The use of isolated whole hearts was pioneered by Oscar Langendorff, who developed the isolated perfused mammalian heart in the year 1895 [51], and since then, it has been widely used and has aided our understanding of the fundamental physiology of the heart. This model has been used in physiological studies of Afib to better understand the mechanisms of this pathology in numerous animals including rabbits [52], sheep [53], guinea pigs [54] and mice [55]. The Langendorff setup enables the delivery of different compounds or the application of catheters. Recently, the use of actual ex vivo human hearts has become a reality and 83 human hearts declined for transplant were resuscitated in the Visible Heart^®^ Laboratory (Minneapolis, MN, USA), showing pulsatile perfusion and evidence of electrocardiogram rhythm, highlighting the significant potential of perfused ex vivo systems previously used with animal hearts [56]. The Langendorff heart-perfused systems offer a powerful testing platform for catheter-based therapies, particularly epicardial devices. The access to a beating heart allows the physical positioning of the catheter to the desired part of the heart, as well as endocardial devices. The Langendorff heart allows complex processes such as the opening of the catheter inside the heart and the localisation of the electrodes to be tested, which is not as easily achieved in vivo. Ablation in perfused systems has been previously reported for ventricular fibrillation [57]; however, nothing has been reported for the atrium. This opens a new avenue of investigation for catheter ablation testing before in vivo studies, obtaining important information regarding ablation size and the effect on the conductive properties of the heart. This model has been reported to be used for monitoring HIFU-induced modifications of electrical conduction in cardiac tissue using real-time fluorescence, aiming to develop appropriate future clinical interventions [58].

Despite the advances and potential of the ex vivo models for Afib studies, these models are complex and challenging to establish and maintain. Typically, the isolated heart models derive from an animal source, which means they do not fully recreate the human condition. The isolated heart is studied in an environment and conditions that do not fully represent those found in the animal or human body, such as the composition of the blood that is commonly substituted with a surrogate buffer [59]. Several molecular processes become less effective after removing the heart from the blood supply, particularly ATP depletion [60]. 

### 2.2. In Vitro Models

**Figure 2:**
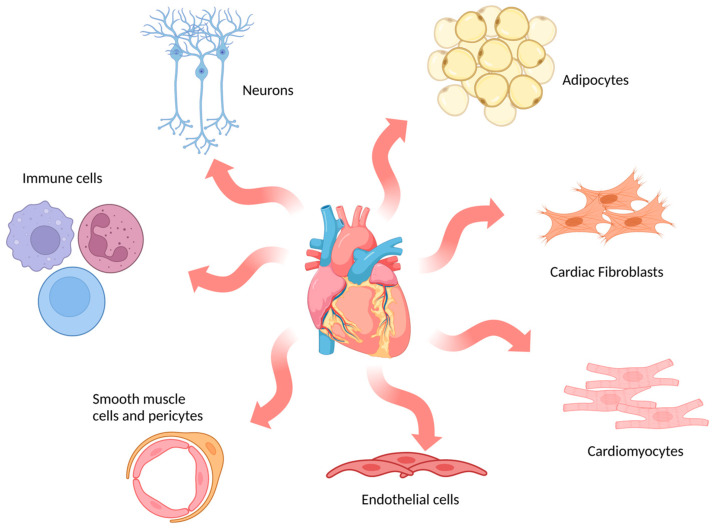
Schematic representation of main cell types in the human heart. The most abundant cell types found in cardiac tissue are indicated in the image: cardiomyocytes, cardiac fibroblasts, endothelial cells, smooth muscle cells and pericytes, immune cells, neurons and adipocytes. (Image created with BioRender.com (accessed on 21 January 2023)).

The adult mammalian heart is composed of multiple cell types (Figure 2), including cardiomyocytes, fibroblasts, endothelial cells, immune cells and mural cells (smooth muscle cells and pericytes), together with adipocytes and neuronal cells [61]. In humans, cardiomyocytes are the dominant cell type by volume (70–80% of the adult heart) and are specialised into atrial or ventricular myocytes which are responsible for the contractile forces of the heart [62]. However, they are not the most numerous cell type. Fibroblasts, which are essential for maintaining the structural, electrical and mechanical features of the heart, are the most abundant cell type in the adult heart [63]. In vitro models encompass a broad range of options that mimic cardiac physiology and pathology to different extents. 

Different cell sources are available for in vitro modelling based on the specific requirements of the study. Primary cardiac cells have been the most commonly used cells in the study of cardiac arrhythmias, typically in monolayer cultures. Primary adult cardiomyocytes with a mature ion channel population and sarcomeric structures present are ideal for electrical [64,65], contractile [66] and calcium dynamic studies [67,68,69], including the effects of ablation parameters on these properties of cardiomyocyte physiology. However, they are complex to maintain in culture and show limited capacity for experimentation, as they can only be maintained for a short period of time after isolation. The isolation process can also be challenging and contamination with other unwanted cell types is common [70]. To overcome these limitations, immortalised cell lines have been developed, normally obtained by introducing an oncogene that sustains active cell proliferation. HL-1 cells are an example of immortalised cell line established from an AT-1 subcutaneous tumour excised from an adult mouse [71] and have been used for the identification of specific proteins and regulatory pathways involved in Afib-related atrial remodelling such as calpain [72], endoplasmic reticulum stress-associated autophagy [73] and more general features of Afib [74]. The immature H9c2 cell line was originally derived from embryonic rat ventricular tissue [75]. These cells are widely used in hypertrophy studies related to heart failure [76,77]. The most recent immortalised cell line is AC16 cells, derived from primary human ventricular cardiomyocytes fused with SV40 transformed human skin fibroblasts [78]. This cell line has been used to study cardiac hypertrophy, oxidative stress, mitochondrial dysfunction and electroporation thresholds [79,80,81,82].

Over the last decade, the emergence of induced pluripotent stem cell (iPSC) technology has greatly advanced our understanding of patient-specific molecular mechanisms of disease and serves as a platform for the development of new therapeutics and drug screening [83]. This technology is based on the ability to reprogram disease-specific patient fibroblasts by forcing the expression of specific transcription factors (Oct4, Sox2, cMyc and Klf4), resulting in a pluripotent state [84]. Subsequently, these pluripotent cells are then differentiated into specific mature cells of interest [85]. This approach has the advantage of maintaining the patient’s complete genetic background and allows the impact of certain key mutations on pathophysiology to be studied. iPSCs have been differentiated into cardiomyocytes through a broad variety of protocols [86] and they have provided new insights into the molecular mechanisms of cardiac diseases [87]. The advantages and limitations of patient-derived cardiomyocytes have been extensively reviewed elsewhere [88].

In vivo tissues are not composed of only one cell type, so the inclusion of two or more cell types in the same system, known as a co-culture, has been shown to offer many advantages when simulating the native tissue [89]. Cardiac tissue has a heterogenous and diverse cell composition. Atrial tissues contain mostly cardiomyocytes and fibroblasts together with other less abundant cell types such as smooth muscle, endothelial and immune cells [61]. It has been reported that essential crosstalk through soluble factors between the cardiomyocytes and fibroblasts is vital for the correct functioning of the cardiac tissue but also can become pathogenic during injury, resulting in a general impairment in electric conduction [90,91]. The ability to include several cell types together in the same space makes the co-culture an attractive model to understand the complex interactions between these cell types and their involvement in Afib [92,93].

Regardless of the selected cell source, cells can be organised and distributed differently to create systems with varying levels of complexity. Models with higher complexity aim to closely resemble the physiological conditions of cardiac tissue but may also require more challenging setups and laborious work. In this review, these models are classified based on their dimensionality into 2D and 3D models.

#### 2.2.1. Adherent Cell (2D) Models

Adherent cell, monolayers or 2D culture is the most common type of in vitro model system. The process is based on cell seeding over a substrate that facilitates growth and proliferation in a single-layer structure. These models are attractive because of their ease of use, i.e., cell growth, manipulation and imaging, which allows for an increased number of replicates and higher throughput. Two-dimensional cultures are well established and reported, with a broad number of studies allowing for results comparison between them. In conventional 2D cultures, the whole cell population has even contact with the nutrients and growth factors present in the medium, which results in homogenous growth and proliferation [94]. Even though the 2D cultures are simplified models, they allowed for discoveries such as the importance of inflammatory processes [95] and the identification of potential drugs such as pioglitazone [96]. Moreover, cell monolayers can be ablated, then the affected area can be assessed, showing the potential for preclinical testing of ablation devices for Afib therapy [82]. Recent developments in iPSC-derived monolayers of cardiomyocytes allowed for a detailed investigation of cell death, induced by PFA and the associated temperature changes [97].

#### 2.2.2. 3D Cell Culture Models

Despite the obvious advantages, 2D systems have certain cell and tissue features that differ appreciably from an in vivo system. One of the most negative examples is the different morphology and lack of tissue organisation found in the native tissue. To overcome these limitations and create a smaller gap between 2D in vitro models and in vivo models, some improvements to the classic 2D system have been developed. Cells respond to geometrical and mechanical patterns present in their environment, and the use of cellular micropatterning provides an opportunity to overcome the lack of such patterns when using the standard tissue culture system [98]. Micropatterning is a technique which allows the position of cells in specific areas of a substrate, enabling the control of cell shape, position and culture architecture [99]. Several methods can be used for this aim, such as the use of polymers as a stamp of the desired microstructure (soft lithography) [100] or using UV through photomasks (photolithography) [101]. Some studies have taken advantage of this technique to create cardiac fibres in vitro to closely mimic the heart tissue environment [102,103]. Important features such as calcium handling, action potential firing and conductional velocities observed in micropatterned cultures makes them more like adult mouse myocardium than traditional monolayer cultures [104]. The use of iPSC together with micropatterning further recapitulates in vivo atrial conduction using a 1D spiral pattern [105], thus overcoming the slow conduction present in other iPSC models of Afib [106]. 

The use of cell monolayers on rigid plastic culture dishes does not allow the cardiac cells to perform physiological contractions, which is the main feature of cardiomyocytes in vivo [107]. In the last few decades, in vitro studies have been trying to overcome this limitation by generating 3D models that can closely represent the physiological behaviour of the cardiac tissue. However, the focus of these models in cardiac research has been primarily on regenerative medicine, specifically the cell replacement lost after cardiac infarction [108]. The number of 3D cell culture systems reported is increasing and the utility of the models is improving. The use of novel 3D in vitro techniques creates new insights into the physiological and pathological processes in heart tissue, making them attractive treatment screening platforms. To date, numerous 3D in vitro models have been developed with different levels of complexity, such as cellular hydrogels and engineered heart tissues (EHTs), organoids and organs-on-a-chip.

##### 3D Hydrogels

**Figure 3:**
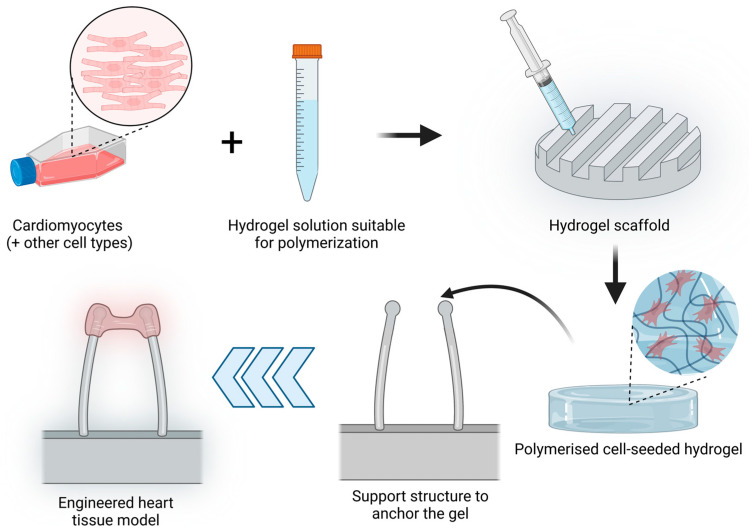
EHT model production process. Cardiomyocytes alone or together with other cell types (such as fibroblasts) are resuspended in a hydrogel solution that can be polymerised. This mix is added to a scaffold with the desired shape and polymerised to obtain a cell-seeded hydrogel. This hydrogel is further placed in a support structure to anchor the gel. In the image, an example of a commonly used support structure is shown to allow the contraction of the cardiomyocyte-seeded hydrogel. (Image created with BioRender.com (accessed on 21 January 2023)).

Hydrogels are crosslinked water-soluble polymers that allow the incorporation of cells embedded into the gel tridimensional structure. The porosity of the hydrogel allows molecules such as growth factors, nutrients or drug loading inside the hydrogel [109] and interact with the seeded cells. Initial 3D hydrogel culture studies were based on single compounds such as collagen type I scaffolds [110] but have been rapidly updated by the addition of several components of the extracellular matrix such as fibrinogen or basement-membrane matrix such as Matrigel [111]. Hydrogels have been widely used to develop EHTs, which are tridimensional muscle constructs made from isolated cardiomyocytes of different animals, human embryonic stem cells (hESCs) and human-induced pluripotent stem cells (hiPSCs), first described in 1997 [110,112]. The elements needed to produce an EHT are cardiac cells, a liquid hydrogel that can be polymerised, a scaffold that will determine the overall EHT shape and a support structure to which the hydrogel will be anchored or attached. The simplified process is described in Figure 3. Over the years, different modifications have been introduced to the support structure, including the use of stretching devices to promote hypertrophy and improved contractile function [113] or two elastic silicone posts to allow auxotonic contractions of the EHT [111].

**Figure 4:**
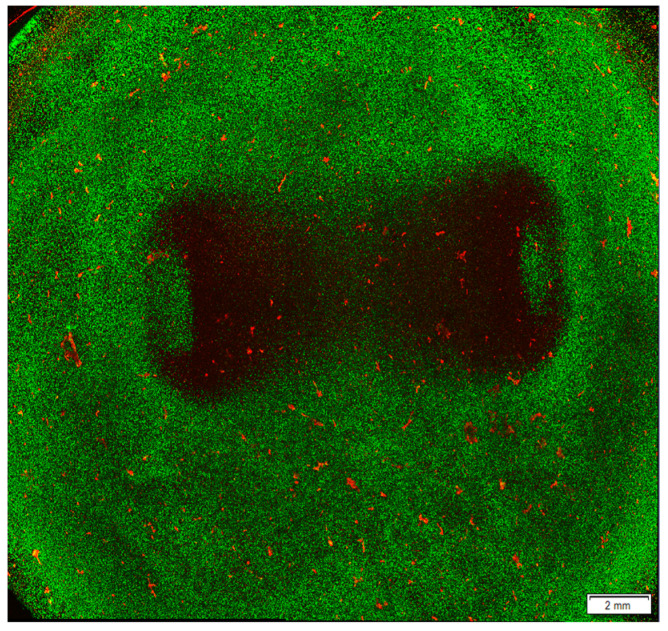
Ablated area after ablation treatment in a hyaluronic acid hydrogel model seeded with cardiomyocytes. AC16 cardiomyocyte cell line was seeded in a hyaluronic acid hydrogel model and treated with 36,000 biphasic pulses of 2 µs duration and 1250 V of input voltage. After ablation, the cells were stained with Life/Dead staining, which labels live cells in green and dead cells in red. The image was obtained using a confocal microscope to visualise the lesion area. Results obtained by the authors of this study and more details on the method used are described in our previous work [79].

EHTs can contract due to the structure of the hydrogel and the interaction between the cardiomyocytes, showing organised sarcomeres and defined beating patterns [114]. These processes, together with electrical stimulation, are essential for the further maturation of the hiPSC-Cardiomyocytes [115]. Recently, the inclusion of other cell types such as fibroblasts has helped create a more accurate representation of the cardiac tissue [116,117]. Furthermore, particularly atrial and ventricular EHTs, can be designed and have shown different features with similarities to those seen in vivo, allowing a highly specific model for the study of cardiac pathologies such as Afib [118]. While EHTs have a great potential for preclinical therapeutic models, as drug screening platforms [119], they are also showing promise as test platforms for ablation-related therapies (Figure 4). The complexity of these models can produce a more accurate result of a given ablation, incorporating a 3D structure as ex vivo models together with viable and healthy cells that can interact and communicate, more representative of the physiological state of the cardiac tissue. Moreover, they keep the relative ease of use and high reproducibility of an in vitro model, avoiding the use of animals for early stages of ablation parameter screening. However, these models are still developing.

Hydrogels can be designed to be temperature-sensitive which would help to predict possible thermal damage risk of a given set of ablation parameters and set up before further animal and clinical studies [120,121]. EHTs based on 3D hydrogels, together with in silico models, can prove beneficial for predicting and planning therapeutic ablation interventions in Afib, as has been shown previously for cancer treatment models [122,123].

##### 3D Organoids

Organoids are tridimensional, multicellular cultures which are capable of self-organisation into complex tissue-like and organ-like structures, supported by an extracellular matrix (ECM). The ability to create these structures is mostly restricted to stem cells, such as pluripotent embryonic stem cells (ESC) or hiPSCs, which can be further matured into the desired cell phenotype. Other cell types that can create organoids are the tissue-resident adult stem cells (ASCs), which are present in adult tissues and show the ability to self-renew and differentiate into other cell types while preserving their tissue specificity [124,125,126]. ASC-based organoids need to be supported by a cocktail of growth factors in the culture media involved in signalling control in vivo tissue conditions.

In recent years, several production methods have been developed, the most common of which is based on cell seeding over or embedded into a matrix such as Matrigel (an ECM protein mix), which provides an appropriate environment. This method allows the monitoring of processes such as cell adhesion, migration and chemotaxis [127]. The use of spinning reactors allows batch production of organoids with larger sizes. Cells are placed in a container which is constantly stirred to avoid cell attachment [128]. The hanging drop method has been used for decades, taking advantage of gravity to induce cell aggregation and assembly in a droplet of medium typically hanging from the lid of a culture dish. This approach was enhanced using hanging drop plates (HDPs), creating an array of spheroids [129]. Other methods employ non-adherent surfaces to cultivate the cells, facilitating them to form aggregates and eventually make spheroids, which is a simple method with high throughput and is more cost-effective than other methods [130,131]. Magnetic nanoparticles have also been employed, relying on the fact that they are taken up by the cultured cells, allowing them to float in the media and favouring its aggregation and production of an ECM [132]. Finally, the use of bioprinting for organoid formation has gained increasing interest in recent years [133]. This technique allows precise control of the shape and distribution of the ECM and organoids, enabling a better representation of the in vivo environment. Bioprinters’ function is based on additive manufacturing, which deposits the desired material layer by layer until reaching the final desired structure [134].

Human cardiac organoids have a huge potential as disease models for heart disorders. They have the capacity to produce spontaneous and induced electrical activity, showing higher conduction velocities than 2D cultures. Cardiac organoids can help study complex electrical arrhythmic processes such as re-entry, hereditary arrhythmias and personalised medicine using hiPSCs. Shinnawi et al. created a cardiac organoid model based on hiPSCs from patients with the arrhythmogenic syndrome, short QT syndrome. This model showed a similar development of re-entry arrhythmia as seen in patients with this syndrome. This study provides evidence of the ability of cardiac organoids to recapitulate disease phenotype and new insights into the mechanisms underlying this arrhythmia syndrome [135]. Organoid cultures show differences in gene expression patterns compared to the in vivo tissues they try to mimic [136]. Organoid cultures show a high heterogeneity of cell phenotypes, containing various clones in different proportions, making studying specific mutations difficult [137]. The lack of vascular and immune systems in organoid cultures limits the representation of the tissue microenvironment [138]; additionally, due to geometrical constraints, device-based intervention testing is limited. Moreover, the use of hiPSC creates ethical regulation challenges [139]. Despite all of these issues, there is significant potential for applications of organoids in the areas of disease modelling and drug screening.

## 3. Conclusions and Future Challenges

Current therapeutic options do not fully address the clinical need for Afib. One potential reason is the lack of appropriate preclinical models to sufficiently support a broad array of test parameters, and treatment options to be assessed and allowing safety testing. Some of the limitations of current models are based on inter-species physiological differences (in vivo models) or the lack of tissue complexity and organ interactions (ex vivo and in vitro models). Due to the complexity of factors involved in Afib, the differences between species and the regulatory processes involved in the use of animals and their organs, the choice of the best preclinical model is a challenge that can be key to the success of the treatment in a clinical setting. There is no perfect Afib model for both disease modelling and treatment screening. To ensure an appropriate model selection, it is crucial to consider the study’s requirements and objectives. A summary of the advantages and limitations of each model is shown in Table 1.

For instance, in PFA testing, a model’s tridimensionality and size are critical factors to determine the ablation zone, whereas these factors may not be as crucial when determining the cell response to an AAD. In vitro models have undergone a revolution in the last number of years, especially with the arrival of hiPSC technology and complex 3D culture systems. Improvements are required to obtain completely mature patient-derived cardiomyocytes, combined with the efforts in optimising 3D models and EHTs such that we can create a useful preclinical model for high-throughput testing of device-based innervations. Including multiple cell types and creating a similar environment to in vivo would provide the influence of the crosstalk between different cell types, which is essential in the heart’s functionality. In the future, these models could be combined with perfusion, and hiPSC-derived heart cells to create the idealised tridimensional in vitro patient-specific heart model that would allow the testing of therapies in advance, allowing clinicians to plan and optimise each patient’s best treatment modality and mitigate risks.

## Figures and Tables

**Table 1:** Classification of research models of Afib with the advantages and limitations of each system.

Model	Implementation	Advantages	Limitations	References
Ex Vivo	Isolated animal whole hearts Heart slices	Controlled environment Highly reproducible experiments Perfusion studies with a complete beating heart Can have human origin Allow physical positioning and opening of a catheter for ablation studies (inside or outside of the heart)	Non-physiological conditions (i.e., blood composition) Whole hearts: complex to maintain for any extended period Heart slices: limited tissue and cell viability; limited supply of human samples	[39,41,46,47,48,49,50,51]
In Vitro	2D adherent models	Rapid screening process Lower costs Wide range of cell options Established protocols Can be human-derived (hiPSC)	Overly simplistic models of the whole heart structure Lacks 3D structure Non-physiological morphology and cell responses Absence of conductivity values for ablation studies	[96,101,102,103,104,105,106,108]
	3D models	3D structure for ablation Potential to measure conductivity Cell–cell and cell–matrix interactions Multiple cell types in physiological disposition (organoids) Can be human-derived (hiPSC), displaying contractile activity	A simple model of the whole heart structure Challenging to produce, manipulate and maintain for extended period Slower screening process Lack of vascular and immune systems	[110,111,112,113,114,115,116,117,118,119,120,121,122,123,124,125,126,129,131,132,135,136,139]

## Data Availability

Not applicable.
